# Host-Pathogen-Venue Combinations and All That Jazz

**DOI:** 10.3201/eid1202.AC1202

**Published:** 2006-02

**Authors:** Polyxeni Potter

**Affiliations:** * Centers for Disease Control and Prevention, Atlanta, Georgia, USA

**Keywords:** Art and science, emerging infectious diseases, Archibald J. Motley, jazz, Nightlife

**Figure:**
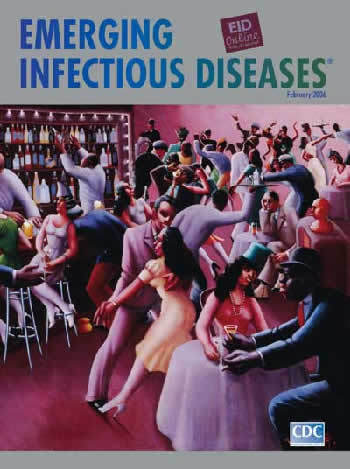
Archibald J. Motley, Jr. (1891–1981). Nightlife (1943). Oil on canvas (91.4 cm × 121.3 cm). The Art Institute of Chicago, Chicago, Illinois, USA. Restricted gift of Mr. and Mrs. Marshall Field, Jack and Sandra Guthman, Ben W. Heineman, Ruth Horwich, Lewis and Susan Manilow, Beatrice C. Mayer, Charles A. Meyer, John B. Nichols, Mr. and Mrs. E.B. Smith, Jr.; James W. Alsdorf Memorial Fund; Goodman Endowment, 1992.89

"To call yourself a New Yorker you must have been to Harlem at least once. Every up-to-date person knows Harlem, and knowing Harlem generally means that one has visited a night club or two," wrote novelist and editor Wallace Thurman, "….The music is good, the dancers are gay, and setting is conducive to joy" (*1*). In the 1920s, nightclubs, bars, and cabarets were much in vogue in most of the western world. In New York, many talented entertainers worked in these clubs, Duke Ellington's orchestra, Cab Calloway's band, Lena Horne, Adelaide Hall. Chicago became a jazz center with more than 100 clubs. "Midnight was like day," wrote poet Langston Hughes describing the city's South Side (*2*). The exotic, glamorous, intoxicated environment of these clubs, which dominated American entertainment for most of the 20th century, was a main source of inspiration to Chicago painter, Archibald Motley.

Motley was born in New Orleans, Louisiana, but his family moved north when he was very young. His mother was a schoolteacher, his father a railroad man, operating a buffet car running on the Michigan Central. Even as a child he sketched scenes and people around him and knew that he wanted to be an artist. The elder Motley mentioned his son's ambitions to Frank W. Gunsaulus of the Armour Institute, a train patron, who paid the youth's first year's tuition at the Art Institute of Chicago. A receptive and eager student, Motley studied under accomplished painter Karl Buehr, who encouraged and advised him: "I want to tell you something, Mr. Motley. I don't want you to ever change your style of painting… please continue it, for my sake" (*3*).

At the Art Institute, Motley indulged his admiration of the old masters, particularly Dutch painter Frans Hals, and was exposed to the work of other American artists (George Bellows, John Sloan, Randall Davey). His graduation in 1918 coincided with the advent of Harlem Renaissance, a cultural movement encompassing the literary, musical, visual, and performing arts and promoting celebration of African identity and heritage. Motley exhibited widely and received many prestigious awards, among them a John Simon Guggenheim Fellowship, which gave him the opportunity to live in Paris for a year. "It is remarkable and beautiful… the way the light travels on the pigmentation of the skin, how gradually the light changes from warm into cool in various faces…. I used to go to the Louvre and study, oh, I studied Delacroix, I studied all the old masters carefully. You know, what we call 'in' painting, the passages of tones" (*3*).

Motley was very productive in Paris. He completed 12 paintings, among them the celebrated Blues, inspired by the local nightclub scene. But he returned to Chicago to exhibit the work. "Artists feel that they're more readily recognized in Europe than they are here in America…. I am staying here in wonderful America. And I love Chicago" (*3*).

"I think that every picture should tell a story," Motley noted (*3*). His narrative paintings, like the work of Frans Hals, peered into the lives of the common people, whom he painted with enthusiasm. But while 17th-century Dutch masters ridiculed drunkenness and warned against the moral laxity of the tavern scene, he viewed social life with affection and offered a glittering rendition of people at play. The club scene with its "total experience" setting provided a perfect backdrop. Extravagantly decorated rooms filled with smoke and spirits called for people to dress up and step out, to escape the reality of postwar depression and social inequity and experience fantasy and luxury in an electrified, unreal environment (*4*). His empathetic portraits and earthy descriptions reflected both his own exuberant love of life and the nightclub scene's whole new view of celebrating: good food, music and dance, and the chance to see and be seen.

"When my grandmother found out that I was playing jazz music," said jazz composer Jelly Roll Morton, "she told me that I had disgraced the family and forbade me to live in the house" (*2*). The music played inside colorful, thickly populated nightclubs all over the United States and spreading around the world, cool jazz, red hot jazz, all manner of jazz, was not always viewed as art form. The music's irregular, sensuous tunes, mixing folk with blues, engaging new instruments, embracing regional sounds, evolved independently in many locations and created an incredible diversity of sounds and styles.

Nightlife, on this month's cover, one of Motley's most celebrated works, is a glimpse of the action at a dance hall in Chicago's Bronzeville neighborhood. Painted during World War II, the picture does not address the dire global circumstances. It focuses instead on a lighthearted moment of gaiety, inside a comfortable establishment, vibrant with the sounds of music, dancing, and conversation. A lively jazz band in the background guides the figures. Diagonal lines indicate sharp syncopated movement amidst free-flowing activity around the dance floor.

The stage is framed with bar paraphernalia, stools, and tables. But the scene is not about the venue. The artist is painting energy and motion, the group dynamic of a community, laughing, gesturing, mingling. The figures are bold but stylized, so the viewer is not distracted by individual features. Body language and overall carriage are harmonious and integrated, and the crowd is engaged and receptive.

Even as Motley focused on the moment's thrill inside a nightclub, he created a microcosm analogous to broader outside reality, an allegory of the world. The stylish crowd socializing and the jazz band orchestrating their movements mirror the group dynamics of microbial populations, swinging to nature's tune in niches they make for themselves. The spontaneity of jazz music and its adaptations to local culture over time around the globe parallel the emergence and export of new diseases from their seedbeds to audiences the world over. A glance at this issue's contents confirms the immense diversity of disease emergence over time and place, from birds with flu and *Helicobacter* infections to drug-resistant HIV strains, from Nipah virus to *Arcobacter*, from dengue to ameba-associated pneumonia. All neatly choreographed to music we cannot yet hear.
